# NEIGHBORHOOD AND HEALTH AMONG CHINESE ADULTS: BEYOND THE URBAN AND RURAL DICHOTOMY

**DOI:** 10.1093/geroni/igz038.304

**Published:** 2019-11-08

**Authors:** Yuekang Li, Yi Wang

**Affiliations:** 1 Washington University in St.Louis, St.Louis, Missouri, United States; 2 The University of Iowa School of Social Work, Iowa City, Iowa, United States

## Abstract

The associations between physical frailty and depressive symptoms among older individuals were established in existing literature. Taking the person-environment perspective, we argue that neighborhood environment could either buffer the stress derived from being physically vulnerable or worsen it by adding another layer of stressors in the environmental context when physical health declined. The objectives of this study are to explore 1) to what extent the neighborhood-level characteristics moderate the relationship between physical frailty and depressive symptoms, 2) if there were rural-urban differences in the moderation effects, and 3) whether some of the environmental factors worked beyond the contextual influences of the rural-urban scope. Using the China Health and Retirement Longitudinal Study 2011 wave, 6,246 individuals ages 60 years and older were included for analyses. Multilevel mixed-effects models were fitted to examine the moderating effects of urbanicity and neighborhood-level socio-economic status (SES) on the relationship between frailty and depressive symptoms among older adults, controlling for individual-level characteristics. Results showed a stronger relationship between deterioration in physical health and depressive symptoms in rural neighborhoods and neighborhoods with lower SES, after controlling for individual-level SES. Also, the moderating effects of the neighborhood-level socioeconomic factors remained after controlling for urbanicity, indicating that neighborhood SES works beyond the rural-urban contexts. Findings from this study demonstrate the important roles of neighborhood socioeconomic characteristics in reshaping and the need to redefining China’s rural-urban dichotomy. The findings also identified neighborhoods with low SES as potential targets for policy and practice to reduce the stress associated with health decline.

